# Cytokine expression in subjects with *Mycobacterium avium* ssp. *paratuberculosis* positive blood cultures and a meta-analysis of cytokine expression in Crohn’s disease

**DOI:** 10.3389/fcimb.2024.1327969

**Published:** 2024-02-13

**Authors:** J. Todd Kuenstner, Qiang Xu, Tim J. Bull, Antonio C. G. Foddai, Irene R. Grant, Saleh A. Naser, Raghava Potula, Peilin Zhang, Ira Shafran, Serhat Emre Akhanli, Svetlana Khaiboullina, Russell Kruzelock

**Affiliations:** ^1^ Department of Biology, Abilene Christian University, Abilene, TX, United States; ^2^ Institute of Infection and Immunity, St. George’s University of London, London, United Kingdom; ^3^ School of Health and Life Sciences, Teesside University, Middlesbrough, United Kingdom; ^4^ School of Biological Sciences, Queen’s University Belfast, Belfast, United Kingdom; ^5^ Burnett School of Biomedical Sciences, University of Central Florida, Orlando, FL, United States; ^6^ Lewis Katz School of Medicine, Temple University, Philadelphia, PA, United States; ^7^ PZM Diagnostics, Charleston, WV, United States; ^8^ Retired, Winter Park, FL, United States; ^9^ Department of Statistics, Muğla Sıtkı Koçman University, Muğla, Türkiye; ^10^ Department of Microbiology and Immunology, University of Nevada, Reno, NV, United States; ^11^ Institute of Fundamental Medicine and Biology, Kazan Federal University, Kazan, Russia

**Keywords:** MAP, Crohn’s disease, bacteremia, inflammatory bowel disease (IBD), autoimmune disease

## Abstract

**Objectives:**

1) Culture *Mycobacterium avium* ssp. *paratuberculosis* (MAP)from blood, 2) assess infection persistence, 3) determine Crohn’s disease (CD) cytokine expression, 4) compare CD cytokine expression to tuberculosis, and 5) perform a meta-analysis of cytokine expression in CD.

**Methods:**

The Temple University/Abilene Christian University (TU/ACU) study had a prospective case control design with 201 subjects including 61 CD patients and 140 non-CD controls. The culture methods included MGIT, TiKa and Pozzato broths, and were deemed MAP positive, if IS900 PCR positive. A phage amplification assay was also performed to detect MAP. Cytokine analysis of the TU/ACU samples was performed using Simple Plex cytokine reagents on the Ella ELISA system. Statistical analyses were done after log transformation using the R software package. The meta-analysis combined three studies.

**Results:**

Most subjects had MAP positive blood cultures by one or more methods in 3 laboratories. In our cytokine study comparing CD to non-CD controls, IL-17, IFNγ and TNFα were significantly increased in CD, but IL-2, IL-5, IL-10 and GM-CSF were not increased. In the meta-analysis, IL-6, IL-8 and IL-12 were significantly increased in the CD patients.

**Conclusion:**

Most subjects in our sample had MAP infection and 8 of 9 subjects remained MAP positive one year later indicating persistent infection. While not identical, cytokine expression patterns in MAP culture positive CD patients in the TU/ACU study showed similarities (increased IL-17, IFNγ and TNFα) to patterns of patients with Tuberculosis in other studies, indicating the possibilities of similar mechanisms of pathogen infection and potential strategies for treatment.

## Introduction

In 1895, Johne and Frothingham first described a chronic diarrheal disease of cattle (now known as Johne’s disease (JD)) (Johne and Frothingham, 1895) and by 1912, Twort et al. identified and cultured the bacterium, now known as *Mycobacterium avium* ssp. *paratuberculosis* (MAP) which causes this disease (Twort et al., 1912). Kennedy Dalziel first speculated in 1913 that MAP also causes Crohn’s disease (CD) (Dalziel, 1913), which he called “chronic interstitial enteritis”. JD is a chronic diarrheal disease of cattle, a wasting disease in sheep and a pathogen in most wildlife species (Over et al., 2011). The demonstration of MAP in the gastrointestinal tissues of patients with CD is notoriously difficult and many investigators have failed to demonstrate the organism but meta-analyses on this topic have concluded that MAP is found in most patients with CD (Feller et al., 2007; Abubakar et al., 2008). The RedHill Biopharma FDA phase II/III clinical trial of combination antimycobacterial therapy for CD supports the thesis that MAP causes CD (Graham et al., 2018).

Recently, a multi-laboratory study employing blind testing of whole blood samples by multiple culture techniques, found widespread viable MAP bacteremia in the 61 CD patients and 140 non-CD control subjects. Nine subjects with a positive Phage assay (4/9) or MAP culture (5/9) were again positive with the Phage assay one year later (Kuenstner et al., 2020). Of the serological methods, the Hsp65 antibody method was best for identifying CD patients from non-CD controls (Kuenstner et al., 2020).

Building upon these findings, our hypothesis is that infection by MAP causes CD in a subset of MAP infected subjects. An objective of this study was to investigate cytokine expression in CD patients and to compare similarities and differences in cytokine expression to other known mycobacterial infections. Thus, we compared cytokine expression patterns in our CD patients to cytokine expression patterns in mycobacterial infections such as JD in cattle and Tuberculosis in humans. Because most of the CD patients and non-CD controls in our Temple University (TU)/Abilene Christian University (ACU) study were positive by at least one culture method (78% and 50%, respectively), CD was used as a proxy for MAP infection. Because MAP is related to *Mycobacterium tuberculosis* (Mtb), we looked for similarities in cytokine expression between tuberculosis and MAP infection. The panel we employed included cytokines that are elevated in tuberculosis, i.e., interferon gamma (IFNγ), IL-2, IL-5, IL-10, IL-17, GM-CSF and TNFα (Jurado et al., 2012; Domingo-Gonzalez et al., 2016; Essone et al., 2019).

After identifying the cytokine profiles in CD patients and non-CD controls, we were interested in whether our work was confirmed by previous work on cytokine expression in CD patients and thus we performed a meta-analysis of data from other CD studies. The largest group of patients with disease in the TU/ACU study had CD, but the non-CD control group included patients with ulcerative colitis (UC) and fewer patients with autoimmune conditions. To perform a meta-analysis of cytokine expression in CD versus non-CD controls, we included the following cytokines which are elevated in CD, i.e., IL-12, IL-17, GM-CSF, IFNγ and TNFα (Ogawa et al., 2012; Vasilyeva et al., 2016; Boucher et al., 2022). We also included IL-22 in our panel, a cytokine which is elevated in IBD, psoriasis, and RA (Zenewicz and Flavell, 2011). The validity of our cytokine analysis in CD was largely confirmed by the meta-analysis and we decided to add our data to the available cytokine data by publishing this meta-analysis.

## Materials and methods

### Study design and participants

The study protocol was reviewed on 20 October 2017 by the Temple University IRB (IRB protocol # 24790). This prospective case-control study included 201 subjects (61 CD patients and 140 non-CD controls). The non-CD control group included 17 subjects with thyroid disease (THY), not otherwise specified, 10 subjects with arthritis (ART), not otherwise specified, 9 subjects with UC, 5 subjects with psoriasis (PSO), 4 subjects with type 1 diabetes mellitus (T1DM), 2 subjects with type 2 diabetes mellitus (T2DM), 2 subjects with rosacea, 2 subjects with irritable bowel syndrome (IBS), 1 subject with asthma (AST), 1 subject with multiple sclerosis (MS), 1 subject with celiac disease (CEL), 1 subject with eczema, 1 subject with lymphoma, and 15 subjects with various combinations of UC, THY, ART, systemic lupus erythematosus (SLE), neurologic disease, AST, and CEL. Please see **Table 1** for a more detailed description of the non-CD control group.

**Table 1:** Health Conditions in the Non-CD controls.

Health Conditions	N	Subject IDs
No reported disease*	69	5001,5012,5014,5017,5019,5021,5036,5037,5038, 5041,5046,5047,5049,5051,5052,5055,5057,5059, 5060,5061,5062,5063,5067,5075,5076,5082,5084, 5087,5088,5095,5097,5098,5102,5104,5105,5110, 5111,5118,5120,5132,5134,5135,5136,5137,5139, 5142,5143,5146,5151,5153,5155,5156,5157,5158, 5159,5160,5161,5162,5165,5167,5175,5176,5180, 5181,5182,5187,5188,5194,5199,5022,5028,5029, 5068,5089,5090,5112,5116,5127,5138,5140,5147, 5149,5170,5177,5178,5183.
Thyroid disease, not further specified	17	5022,5028,5029,5068,5089,5090,5112,5116,5127, 5138,5140,5147,5149,5170,5177,5178,5183.
Arthritis, not further specified	10	5044,5070,5072,5073,5080,5085,5091,5108,5130, 5152.
Ulcerative colitis (UC)	9	5003,5013,5040,5045,5101,5103,5117,5131,5171.
Psoriasis	5	5024,5119,5122,5158,5191.
Type 1 diabetes mellitus (T1DM)	4	5025,5042,5077,5184.
Type 2 diabetes mellitus (T2DM)	2	5168,5185.
Rosacea	2	5197,5201.
Irritable bowel syndrome	2	5107,5172.
Asthma	1	5083
Multiple sclerosis (MS)	1	5164
Eczema	1	5179
Celiac disease	1	5169
Lymphoma	1	5144
Combinations of UC,THY,ART,PSO,SLE, NEU,AST, & CEL.	15	5004,5015,5031,5043,5054,5069,5092,5113,5114, 5125,5128,5173,5189,5193,5200.
All subjects in the Non-CD control group	140	

*The study subjects were asked whether they had autoimmune diseases but not whether they had Hypertension, cardiovascular or peripheral vascular disease or cancer.

The first 159 subjects in the study were recruited by Dr. Ira Shafran, a gastroenterologist in Winter Park, Florida. In addition, 42 of the subjects were recruited from the Human Paratuberculosis Foundation website (www.humanpara.org) and the phlebotomy of the second group was performed at a site in New York City. A single blood collection was performed on 192 subjects during May through August 2018. For 9 of the subjects, a second blood sample was obtained in August 2019 to determine if they had a persistent MAP infection. Selection of these subjects was based on an initial positive phage assay (4/9) or MAP culture (5/9) and for their availability to donate a second sample one year later in Philadelphia. One of these subjects had chronic thyroiditis and IBS, three had chronic thyroiditis, four subjects were asymptomatic and healthy, and one had IBS. The subjects completed a consent form and questionnaire about their medical history. All subject information was kept confidential in accordance with standard medical practice.

### Procedures

At enrollment, blood samples were collected from the 201 participants in EDTA blood collection tubes. Peripheral blood leukocytes (PBLs)/buffy coat specimens were prepared in the Potula laboratory from the whole blood samples and shipped to each of the laboratories performing the cultures (Bull and Grant laboratories in London and Belfast, UK, respectively, and Naser laboratory in Florida, USA). The courier delivery time to the Bull and Grant laboratories was subject to inconsistently applied international customs regulations and thus ranged from 3 to 7 days while the courier delivery time to the Naser laboratory was one day. The plasma from the blood samples was collected and frozen at -80 °C until serologic or cytokine testing was performed. All samples were identified by a study number and the laboratory scientists were blinded to all clinical information, diagnoses and personal identifiers.

### Plasma cytokine measurement and analysis

Cytokines IL-1β, IL-12p70, IL-10, IL-2, IL-4, TNFα, IFNγ, and IL-6 in participant plasma samples were measured using Simple Plex Cytokine Screening Panel cartridges (SPCKE-PS-003426, ProteinSimple) with the Ella Next Generation ELISA system (ProteinSimple). The lower and upper limit of quantification were 0.21 and 840 pg/ml for IL-1β, 0.46 and 2.7 pg/ml for IL-12p70, 0.46 and 5530 pg/ml for IL-10, 0.64 and 990 pg/ml for IL-2, 0.32 and 1290 pg/ml for IL-4, 0.17 and 4000 pg/ml for IFNγ, 0.3 and 1160 pg/ml for TNFα, and 0.28 and 2652 pg/ml for IL-6. Cytokines IL-5, IL-17A, IL-8, IL-33, and GM-CSF in participant plasma samples were measured using custom made Simple Plex Cytokine Screening Panel cartridges (SPCKE-PS-007313, ProteinSimple) with the Ella Next Generation ELISA system (ProteinSimple). The lower and upper limit of quantification were 0.45 and 4290 pg/ml for IL-5, 1.05 and 10000 pg/ml for IL-17A, 0.19 and 1840 pg/ml for IL-8, 1.31 and 1140 pg/ml for IL-33, and 0.70 and 6730 pg/ml for GM-CSF. Cytokine IL-22 in participants’ plasma samples was measured using custom made Simple Plex Cytokine cartridges (SPCKE-PS-001529, ProteinSimple) with the Ella Next Generation ELISA system (ProteinSimple). The lower and upper limit of quantification for IL-22 were 6.64 and 63300 pg/ml, respectively.

The Ella Next Generation ELISA system is highly automated and requires no manual washes. Briefly, participant plasma samples were spun down at 4°C, 10000g for 5 min. Then, 28 μl of plasma samples were added to 28 μl of diluent SD13 and mixed well. After a quick spin, 50 μl of diluted samples were added into the individual sample inlets of the cartridge. After adding samples to all the sample inlets of the cartridge, 1000 μl of wash buffers were added into the corresponding inlets. After all inlets were filled with samples or wash buffers, a run was initiated by using Simple Plex Runner software. All immunoassay processes (including prime system, flow samples and splitting into channels, sample incubation, wash, rehydrate and flow secondary antibody, wash, rehydrate and flow streptavidin dye conjugate, incubate, wash, scan) are processed automatically. Following a 70-minute run, cytokine concentration results in pg/ml of each sample (triplicate results for IL-22 (single analyte cartridge SPCKE-PS-001529) and duplicate results for all other cytokines (multi analytes cartridges SPCKE-PS-003426 and SPCKE-PS-007313)) are generated using cartridge built-in standard curves. Raw signal data (relative fluorescence units, RFUs), mean signal values, standard deviation, and coefficient of variance (CV) for each Glass Nano Reactor (GNR) value are also provided for further analysis.

### MGIT, TiKa and Pozzato cultures and phage amplification assay

The MGIT (Naser Laboratory), TiKa (Bull Laboratory), Pozzato (Grant Laboratory) cultures and the phage amplification assay (Grant Laboratory) were performed as described previously and were deemed MAP positive, if IS900 PCR positive (Kuenstner et al., 2020).

#### Controls for TiKa culture and PCR

In total, 201 samples were received in nine batched deliveries over a 5-month period (2018). Each batch was processed along with two reagents only controls (using a sterile water aliquot as a base) which were cultured alongside samples in TiKa-MGIT medium and on initial solid culture plates. At the end of the experiment (this ended up being 24 months in many cases) all control tubes were harvested and concentrated by centrifugation at 1,700xg for 15 mins then extracted for DNA alongside all other samples. DNA extractions also included water process negative controls that were processed in parallel with samples. All PCR runs included 1) extractions from culture process negative media controls 2) extractions from DNA process negative controls 3) PCR reagent only controls. For samples that were sub cultured, a negative tube/plate was always included and checked for colonies/growth alongside a positive control (a human MAP strain from Bull’s collection). None of the controls grew organisms and all DNA extractions and reagent controls tested by IS900 PCR were negative. As mentioned above, small aliquots of Grant culture samples were additionally sub-cultured onto Blood Agar to check for fast growing contaminants.

#### Controls for Pozzato culture

New (i.e., previously unused) and pre-sterilized (autoclaved) glass culture tubes were used for all samples and controls. Negative growth control was an un-inoculated Pozzato broth incubated alongside each batch of test samples. Positive growth control was a Pozzato broth inoculated with 100 µl of 10^-4^ dilution of 4-week-old MAP culture incubated alongside each batch of test samples.

#### Controls for MAP phage assay

Negative control was 1 ml 7H9/OADC/2 mM CaCl_2_ broth processed through phage assay along with samples. The presence of <10 plaques for the negative control was necessary to confirm the efficacy of FAS treatment to kill extraneous D29 phages. Positive control was 1 ml 10^-5^ dilution of 4-week-old MAP culture in 7H9/OADC/2 mM CaCl_2_ broth processed through phage assay, which should yield ~100 plaques after overnight incubation. Negative growth control for plaque PCR was molecular grade water in place of template DNA. Positive growth control for plaque PCR was 5 µl DNA extracted from 10^-4^ dilution of 4-week-old MAP culture by boiling for 25 min and then brief centrifugation.

### MAP antibody assay (Potula laboratory)

Plasma samples for each patient were assayed using the IDEXX MAP ab Test for detection of antibody to MAP in bovine serum, plasma, and milk. This test was adapted for human use as described previously (Bernstein et al., 2004). Human plasma controls optical density (OD) values were used to calculate sample/positive (S/P) ratios and interpret the assay.

### Hsp65 Antibody Assay (Zhang Laboratory)

Hsp65 antibody from the blood was measured by direct ELISA assays described previously (Zhang et al., 2019). Recombinant Hsp65 from *Mycobacterium avium* subspecies *hominissuis* (MAH) was produced at GenScript Corp (https://www.genscript.com/) through contract work and used to coat the 96-well plate.

### Data analysis and statistical methods

In the cytokine variables, there are several extreme values, which have a huge effect on the distributions of the variables. To handle very skewed data distributions, log-transformation is a widely used technique in biomedical, medical, and psychological research (Limpert et al., 2001; Feng et al., 2014). To minimize the impact of outliers, logarithmic transformation (log(x+c)) was applied prior to conducting appropriate statistical analyses. Because the cytokine variables have numerous zero values, a constant (c=1) was added to each value. Log-transformation has been used in prior articles on CD (Meuwis et al., 2013; Boucher et al., 2022).

The summary of the descriptive data is presented as numbers (percentages) for categorical variables. Continuous variables are presented as mean ± standard deviation or median and interquartile ranges (1^st^ quartile – 3^rd^ quartile) for skewed data. To investigate whether the continuous variables are normally distributed or skewed, the Shapiro’s Wilk test was used (**Table 2**). Independent samples t-tests or non-parametric Mann-Whitney U-tests were adopted to compare the continuous variables between two groups. Pearson’s chi-square was used for the categorical variables. The analyses were performed using the R software (version 4.0.0). The results were considered significant at a level of p < 0.05.

**Table 2:** Shapiro’s Wilk test p-values for Crohn’s disease (CD) data set.

Variables	p-value for Non-CD group	p-value for CD group
**Age (years)**	<0.001	0.005
**Log (IL-1β pg/ml)**	<0.001	0.037
**Log (IL-2 pg/ml)**	<0.001	<0.001
**Log (IL-4 pg/ml)**	<0.001	<0.001
**Log (IL-5 pg/ml)**	<0.001	<0.001
**Log (IL-6 pg/ml)**	<0.001	<0.001
**Log (IL-8 pg/ml)**	0.886	0.224
**Log (IL-10 pg/ml)**	<0.001	<0.001
**Log (IL-12p70 pg/ml)**	<0.001	<0.001
**Log (IL-17A pg/ml)**	<0.001	0.049
**Log (IL-22 pg/ml)**	<0.001	<0.001
**Log (IL-33 pg/ml)**	<0.001	<0.001
**Log (IFNγ pg/ml)**	<0.001	<0.001
**Log (TNFα pg/ml)**	0.074	<0.001
**Log (GM-CSF pg/ml)**	<0.001	<0.001

• H_0_: Data is normally distributed.

• H_*A*_: Data is not normally distributed.

The meta-analysis was done using the Temple University (TU)/Abilene Christian University (ACU) (Kuenstner et al., 2020), Vasilyeva (Vasilyeva et al., 2016) and Boucher (Boucher et al., 2022) data. Data was requested from the authors of the Ogawa (Ogawa et al., 2012) study, but none was provided. The cytokines studied in common by the TU/ACU, Vasilyeva and Boucher studies are IL-1β, IL-2, IL-4, IL-5, IL-6, IL-8, IL-10, IL-12, GM-CSF, IFNγ and TNFα. To determine the cytokine levels, the three studies in the meta-analysis used different analytical methods with different analytical sensitivities and reference ranges.

One of our goals was to compare these three studies which reported cytokine levels in CD patients and controls. Although a meta-analysis requires several independent studies of the same subject, Ryan (Ryan and Cochrane Consumers and Communication Review Group, 2016) pointed out that a minimum of two studies are sufficient for a meta-analysis. Vasilyeva’s data has 105 subjects with 58 adults (52 with CD) and 47 children (37 with CD). In the TU/ACU and Boucher studies, the age of the patients is between 16 and 87, and only the adults in Vasilyeva’s study are included for the meta-analysis for consistency of subject comparison of the three studies.

Because the cytokines are highly skewed in both the TU/ACU and Vasilyeva data sets, logarithmic transformation (*log(x+c)*) was applied prior to conducting appropriate statistical analyses. The data set in Boucher’s study were already log-transformed, so that it was not necessary to apply logarithmic transformation, but an additional constant value, “*c*” was included prior to conducting the meta- analyses for consistency. The R software package (version 4.0.0), and the “*metacont*” and “*forest*” functions “*meta*” were used for all analyses.

Several parameters should be determined before performing a meta-analysis. One parameter is the “summary measurements” that have the selections as “Mean Difference (MD)”, “Standardized Mean Difference (SMD)” and “Ratio of Means (ROM)” to be used for pooling of studies. Deeks et al. (Deeks et al., 2019) pointed out that mean and standard deviation summaries can give misleading results when studies are small, and the data is skewed or non-normally distributed. Hedges et al. (Hedges et al., 1999) and Friedrich et al. (Friedrich et al., 2008) recommended that the ratio of means is the right approach for log transformed data. Therefore, the ratio of means was used for summary measurements of the pooled studies.

## Results

The TU/ACU study included 61 patients with CD (33 female, 28 males) and 140 subjects without CD (82 female, 58 males) (**Table 3**). CD is not significant for gender variables (p=0.664) (**Table 3**). Patients with CD were significantly younger than subjects without CD (p=0.015) (**Table 3**). The median (1^st^ quartile - 3^rd^ quartile) age of the non-CD group was 57.5 (37-66), whereas the median age of the CD group was 47 (35-60) (**Table 3**).

**Table 3:** Summary of variables and data for the TU/ACU study.

Variables	Non-CD (n=140)	CD (n=61)	p-values
**Gender**			0.664
**Female**	82 (58.6%)	33 (54.1%)	
**Male**	58 (41.4%)	28 (45.9%)	
**Age (years)**	57.5 (37 – 66)	47 (35 – 60)	**0.015**
Culture techniques			
**TiKa culture (+)**	42 (30.2%)	22 (36.7%)	0.466
**MAP Phage assay (+)**	85 (60.7%)	28 (45.9%)	0.073
**Pozzato culture (+)**	89 (63.6%)	35 (57.4%)	0.501
**MGIT culture (+)**	21 (15%)	15 (24.6%)	0.153
Cytokine variables with log (x+1) transformation			
**Log (IL-1β pg/ml)**	1.33 (0.81 – 1.92)	1.39 (1.05 – 2.1)	0.126
**Log (IL-2 pg/ml)**	0.02 (0 – 0.08)	0.03 (0 – 0.12)	0.293
**Log (IL-4 pg/ml)**	0 (0 – 0.04)	0 (0 – 0.04)	0.589
**Log (IL-5 pg/ml)**	0.22 (0.14 – 0.34)	0.18 (0.1 – 0.29)	0.061
**Log (IL-6 pg/ml)**	0.89 (0.7 – 1.11)	0.97 (0.76 – 1.5)	0.061
**Log (IL-8 pg/ml)**	2.93 ± 0.72	3.04 ± 0.87	0.409
**Log (IL-10 pg/ml)**	0.97 (0.85 – 1.08)	1.02 (0.87 – 1.13)	0.171
**Log (IL-12p70 pg/ml)**	0.31 (0.13 – 0.44)	0.37 (0.22 – 0.51)	0.129
**Log (IL-17A pg/ml)**	0.55 (0.35 – 0.75)	0.63 (0.47 – 0.88)	**0.037**
**Log (IL-22 pg/ml)**	1.66 (0.23 – 2.48)	2.07 (1.16 – 2.81)	0.065
**Log (IL-33 pg/ml)**	0.37 (0.18 – 0.6)	0.39 (0.19 – 0.67)	0.564
**Log (IFNγ pg/ml)**	0.39 (0.28 – 0.51)	0.5 (0.36 – 0.9)	**0.002**
**Log (TNFα pg/ml)**	2.16 (2.02 – 2.35)	2.32 (2.01 – 2.69)	**0.019**
**Log (GM-CSF pg/ml)**	0.55 (0.41 – 0.84)	0.61 (0.43 – 1.11)	0.274

Data expressed as mean ± SD or median (1^st^ quartile – 3^rd^ quartile) for continuous variables or n (%) for categorical variables.

Log (IL-8 pg/ml) compared using parametric independent samples t-test.

Nonparametric Mann Whitney U test was used for the other numerical variables.

TiKa, MGIT, Pozzato cultures and MAP Phage assay compared using Chi-square tests.

Bold p-values indicate statistical significance at α=0.05.

As previously reported (Kuenstner et al., 2020), viable MAP bacteremia was reported in most of the study subjects. However, no significant relationships exist between CD and Non-CD for any of the culture techniques, see the p-values (> 0.05) in **Table 3**.

The use of positive and negative process controls in the Bull, Grant and Naser laboratories makes laboratory contamination unlikely as the source of the MAP organisms in the subjects. Furthermore, genomic sequencing using assembled contigs of MAP isolates from subculture from seven CD patients (Subjects 5010, 5065, 5078, 5093, 5126, 5141 and 5186) clustered in with MAP type C phylogeny but showed genetic diversity. By the same method, MAP isolates from subject 5186 which grew in the Grant and Bull laboratories were virtually identical. These findings are also consistent with subject infection by MAP rather than laboratory contamination (unpublished results from Bull laboratory).

The Hsp65 Ab was a better test for discriminating the CD cases from the non-CD controls than the modified IDEXX MAP Ab test. The sensitivity and specificity of the Hsp65 Ab was 0.574 and 0.607, at a cutoff of 0.74, respectively, confirming the finding reported in our earlier study (Kuenstner et al., 2020).

**Figure 1:**
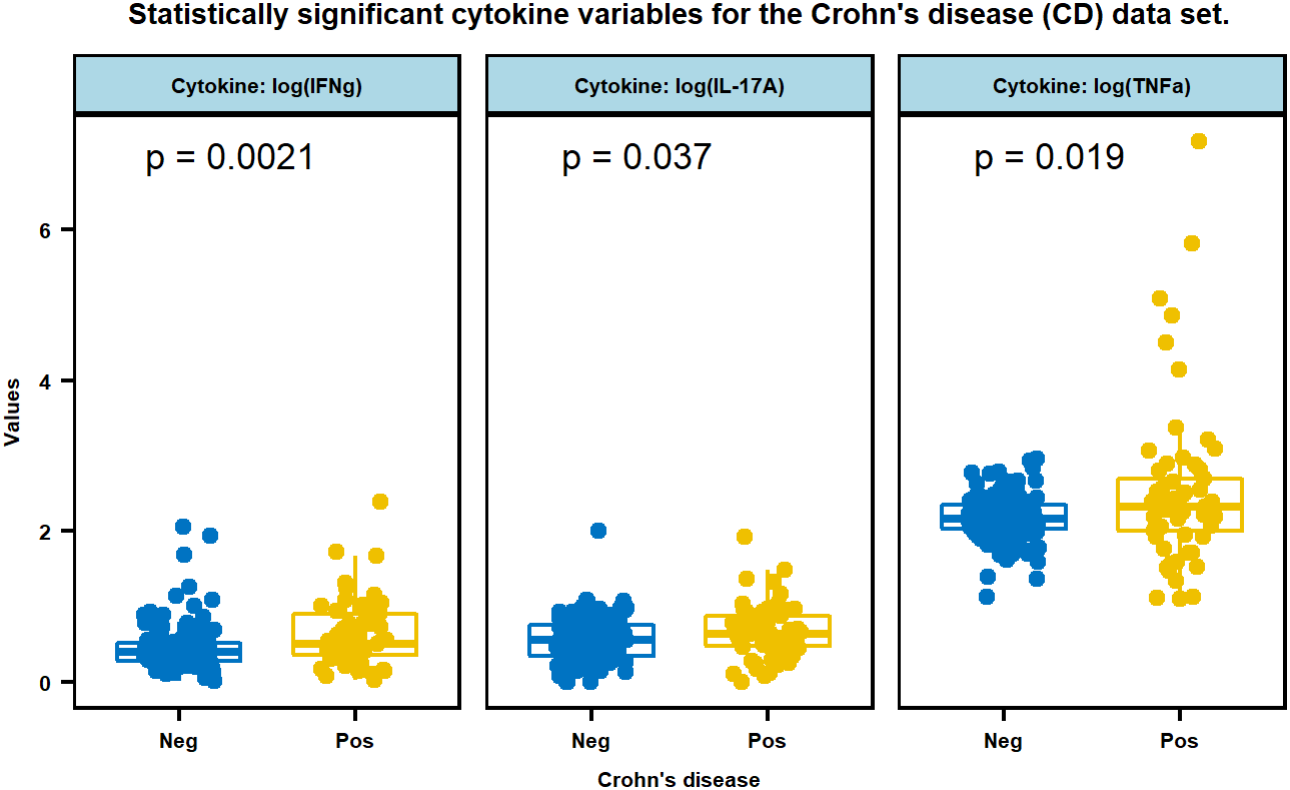
Multiple boxplots of three cytokines, IFNg, IL-17A, and TNFa that are statistically significant between CD positive and negative subjects. Cytokine values were transformed by log (x+1).

In the TU/ACU study, except for IL-8, the log-transformed cytokines are distributed in a nonparametric fashion (**Table 2**). Among all these variables, IFNγ, TNFα and IL-17A are significantly different between the CD and Non-CD groups (**Figure 1**), and the p-values and the statistical summary are given in **Table 3**. There is no effect of CD on the other cytokines. The CD patients with MAP infection in the TU/ACU study had elevated (IFNγ), TNFα and IL-17A like tuberculosis patients, but they did not have elevated IL-2, IL-5, IL-10 and GM-CSF typically reported in tuberculosis patients.

**Table 4** includes the results of the meta-analysis for the three pooled studies. The first two columns are the ratio of means of two groups (CD and controls) for the TU/ACU, Vasilyeva and Boucher studies. The next columns are statistical values for heterogeneity including (*I* ^2^) statistics, which is the strength of evidence for heterogeneity and Cochran’s Q test’s p-value, which examines whether the pooled studies are equally effective (*H*_0_) or not (*H_A_*). Deeks et al. (Deeks et al., 2019) provided a rule of thumb for the interpretation of *I* ^2^ statistics as:

- 0% to 40%: might not be an important level of inconsistency,
- 30% to 60%: may represent moderate heterogeneity,
- 50% to 90%: may represent substantial heterogeneity,
- 75% to 100%: considerable heterogeneity.

**Table 4:** Meta-analysis results for the cytokine variables with log (x+1) transformation.

Variables (pg/ml)	ROM (*)			Heterogeneity (**)		Fixed effect models		Random effect models	
	*TU/ACU*	*Vasilyeva*	*Boucher*	*I* ^2^	*p – val*	*ROM*	*p – val*	*ROM*	*p – val*
Log (IL-1β)	1.1235	1.4730	1.0226	92%	<0.001	1.0694	0.002	1.1863	0.125
Log (IL-2)	1.5833	0.9678	0.8231	85%	0.001	0.9151	<0.001	0.9724	0.827
Log (IL-4)	0.8224	0.9379	1.0086	0%	0.373	1.0061	0.549	0.9911	0.769
Log (IL-5)	0.8195	1.0173	1.0389	54%	0.112	1.0304	0.081	1.0292	0.117
Log (IL-6)	1.2029	1.1306	1.1096	0%	0.502	1.1209	<0.001	1.1209	<0.001
Log (IL-8)	1.0199	1.1206	1.0582	0%	0.443	1.0573	<0.001	1.0573	<0.001
Log (IL-10)	1.0411	0.9874	1.0635	49%	0.141	1.0134	0.387	1.0218	0.355
Log (IL-12)	1.2378	1.1857	1.0430	32%	0.228	1.0480	0.003	1.1031	0.162
Log (GM-CSF)	1.1998	1.2331	0.9676	78%	0.010	0.9868	0.531	1.0985	0.275
Log (IFNγ)	1.5008	1.1158	1.0212	94%	<0.001	1.0283	<0.001	1.1741	0.134
Log (TNFα)	1.0764	0.9987	1.0260	49%	0.140	1.0263	0.038	1.0287	0.122

* ROM: Ratio of means (ROM>1 indicates an increase in the CD patients in comparison to the non-CD controls).

** *I* ^2^ is the heterogenity statistics and its p-value.

Nevertheless, they stated that researchers may need to avoid meta-analysis results when there is large heterogeneity. For the last columns, fixed effect model statistics provide a ratio of mean values and the p-value for examining the difference between the CD and non-CD groups for the pooled studies. Ryan (Ryan and Cochrane Consumers and Communication Review Group, 2016) indicated that the decision between the fixed-effect model or the random-effects model is a researcher’s judgement. On the other hand, he also advised that, “*if the random- effects model and the fixed-effect model produce substantially different pooled estimates then this is an excellent indication of heterogeneity, and the random-effects model is the preferred model. If the two models yield similar pooled estimates, then the fixed-effect model is preferred, because usually it will have a narrower confidence interval; that is, it is more precise than the random-effects model.”* Our interpretation for the meta-analysis results in **Table 4** is based on the argument above.

**Figure 2:**
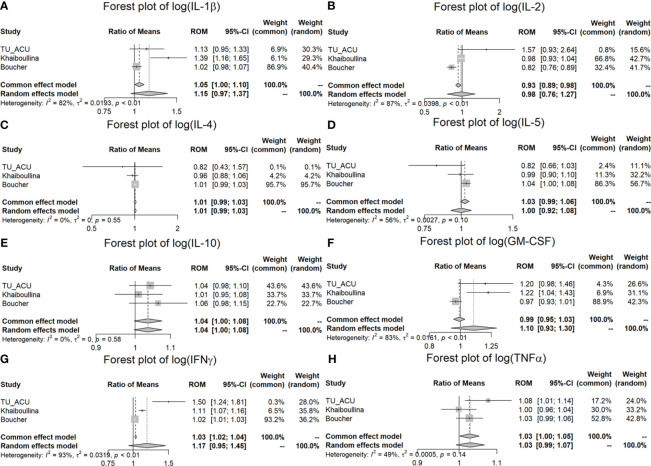
Meta-analysis of plasma cytokine differences between CD patients and controls in three studies. **(A)** Forest plot of log (IL-1β); **(B)** Forest plot of log (IL-2); **(C)** Forest plot of log (IL-4); **(D)** Forest plot of log (IL-5); **(E)** Forest plot of log (IL-10); **(F)** Forest plot of log (GM-CSF); **(G)** Forest plot of log (IFNγ); **(H)** Forest plot of log (TNFα).

According to the pooled results of the meta-analysis in **Table 4** and **Figure 2**, the log (IL-1β), log (IL-2), log (GM-CSF) and log (IFNγ) variables have considerable heterogeneity (*I* ^2^ = 92%; p < 0.001, *I* ^2^ = 85%; p = 0.001, *I* ^2^ = 78%; p = 0.01 and *I* ^2^ = 94%; p < 0.001) among the three studies, and the random effects model shows there is no significant difference between the CD and control groups. Next, the log (IL-5) and log (IL-10) variables show substantial heterogeneity in the three studies, but their p-values indicate that there is no such evidence to reject the pooled studies to be equally effective (*I* ^2^ = 54%; p = 0.112 and *I* ^2^ = 49%; p = 0.141). There is no statistically significant difference between the CD and control groups based on both the results of the fixed and random effects models for these two cytokines. The log (IL-12) and log (TNFα) variables show moderate heterogeneity *I* ^2^ = 32%; p = 0.2228 and *I* ^2^ = 49%; p = 0.1397), and there are differences between the CD and control groups based on the fixed effect model (p = 0.003 and p = 0.038) for the log (IL-12) and (TNFα) variables, respectively. On the other hand, the log (IL-4), log (IL-6) and log (IL-8) variables show homogeneous results for both studies (see their *I* ^2^ (= 0%) statistics and p-values (>0.1) in **Table 4**). According to the fixed and random effect model results, we found a statistically significant difference between the CD and control groups for log (IL-6) (p < 0.001) and log (IL-8) (p < 0.001) variables, but not for log (IL-4) (p = 0.769). Although log IL-4 and log IL-10 variables have homogeneous results (p = 0.373 and p = 0.141, respectively), there is no significant difference between the CD and control groups for the pooled studies (p = 0.549 and p = 0.387, respectively).

## Discussion

Viable MAP bacteremia was detected in most of the TU/ACU study subjects in both the CD and the non-CD control groups by several methods. We were surprised by the finding that so many of the non-CD control subjects in our study had positive MAP blood cultures. Following further reflection on this surprising finding, we note that our observation in human subjects resembles the findings of a recent study on MAP bacteremia in the cattle population. In this study, MAP bacteremia was noted in asymptomatic and subclinical cattle without JD (Greenstein et al., 2021).

Our observation of MAP bacteremia in so many asymptomatic subjects without active disease also resembles the situation with infection by Mtb. Following our discovery of MAP bacteremia detected by a MAP phage assay, Verma et al. showed that Mtb bacteremia could be detected by a Mtb phage assay in the blood of subjects with latent and active tuberculosis infection (Verma et al., 2020). In addition, the global prevalence of latent tuberculosis was 23.67% in 2019, 10 million became sick and 1.5 million died from tuberculosis (Ding et al., 2022). Thus, a minority of subjects with latent infection develop active infection.

Because most of the CD patients in the TU/ACU study were positive by at least one culture method (78%), CD was used as a proxy for MAP infection and for the comparison of cytokine expression between MAP infection and Mtb infection. Like tuberculosis patients, the CD patients in the TU/ACU study had elevated IFNγ, IL-17 and TNFα (see **Figure 1**; **Table 3**). Increased IFNγ and IL-17 have also been reported in MAP infected cattle with JD (Dudemaine et al., 2014). However, in contrast to tuberculosis patients, the CD patients in the TU/ACU study did not have statistically significant increased IL-2, IL-5, IL-10, and GM-CSF (see **Table 3**).

**Figure 3:**
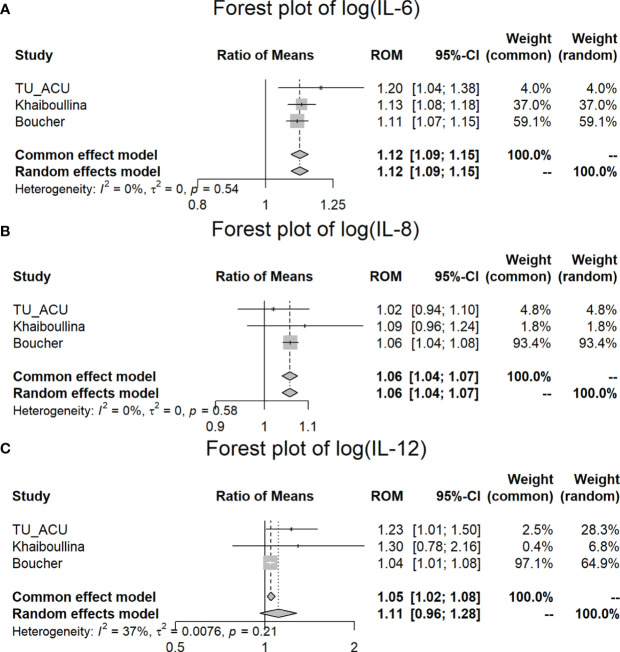
Meta-analysis of plasma IL-6, IL-8, and IL-12 differences between CD patients and controls in three studies. **(A)** Forest plot of log (IL-6); **(B)** Forest plot of log (IL-8); **(C)** Forest plot of log (IL-12).

In the meta-analysis, we found that IL-6 (*p* < 0.001), IL-8 (*p* < 0.001) and IL-12 (*p* = 0.003) were significantly increased in the CD patients in comparison to the controls for all three studies (ROM values are all greater than 1) (**Table 4**; **Figure 3**). TNFα is significantly increased (*p* = 0.038) in the TU/ACU and Boucher studies (ROM>1), but there is a slight decrease (almost equal, ROM=0.9987≈1) in the Vasilyeva study (**Figure 2**). The differences in the cytokines in the three studies probably reflect that 1) the TU/ACU study included non-CD controls some of whom had other autoimmune diseases, 2) the Vasilyeva study included a comparatively small non-CD control group of 23 adults and 3) that the Boucher study included a comparatively large number of unrelated healthy controls.

The non-CD control group of the TU/ACU study included 4 patients with T1DM and 1 patient with MS, diseases which are also associated with MAP infection (Masala et al., 2015; Cossu et al., 2019). The inclusion of other diseases associated with MAP would be expected to result in a less robust non-CD control group.

The cytokine expression patterns reported in the TU/ACU study were not performed on treatment naïve CD patients and thus the therapy and activity of the disease affected the cytokine expression in this study. Although cytokine expression in CD is unlikely to be useful for diagnosis, it may provide insights into the pathophysiology of CD.

Future investigations of the role of MAP in human infection should include 1) long term studies to determine if asymptomatic subjects with MAP infection will eventually develop active CD or other diseases, 2) a comprehensive examination of which host factors affect the suppression or expression of disease in MAP infected subjects, and 3) studies of the MAP bacterium in human infections examining MAP virulence factors.

In conclusion, the linking of highly upregulated IL-17 responses with CD patients in this study confirms patterns of immunoreactivity previously seen in naïve untreated (pediatric) CD patients (Ashton et al., 2021). That similar reactivities are seen within clinical, subclinical, and early stages of MAP infected animals (DeKuiper et al., 2020), links this key pro-inflammatory cytokine’s involvement in the development and maintenance of both diseases. It is currently unknown if IL-17 is hyper-inflammatory or driving chronic inflammation in response to chronic invasive bacteria. However, MAP is a proven long term persistent intracellular pathogen which, post-infection, is capable of driving and maintaining IL-17 responses. The connection made in this study between CD patients having both chronic MAP bacteremia and IL-17 immunoreactivity and some of the similarities in cytokine expression in CD and Tuberculosis reinforces the proposed role of MAP in the pathophysiology of CD.

## Data availability statement

The original contributions presented in the study are included in the article/supplementary material. Further inquiries can be directed to the corresponding author.

## Ethics statement

The study protocol was reviewed on 20 October 2017 by the Temple University IRB (IRB protocol # 24790). The studies were conducted in accordance with the local legislative and institutional requirements. The participants provided their written informed consent to participate in this study.

## Author contributions

JK: Investigation, Methodology, Writing – original draft, Writing – review & editing, Conceptualization, Data curation, Formal Analysis, Funding acquisition, Project administration, Resources, Supervision. QX: Data curation, Formal Analysis, Funding acquisition, Investigation, Methodology, Project administration, Resources, Writing – review & editing. TB: Data curation, Investigation, Methodology, Writing – review & editing. AF: Data curation, Investigation, Methodology, Writing – review & editing. IG: Data curation, Investigation, Methodology, Writing – review & editing. SN: Data curation, Investigation, Methodology, Writing – review & editing. RP: Data curation, Investigation, Methodology, Writing – review & editing. PZ: Data curation, Investigation, Methodology, Writing – review & editing. IS: Data curation, Investigation, Methodology, Writing – review & editing. SA: Data curation, Formal Analysis, Investigation, Methodology, Writing – review & editing. SK: Data curation, Methodology, Writing – review & editing. RK: Conceptualization, Investigation, Methodology, Project administration, Supervision, Writing – review & editing.
